# Defense against predators incurs high reproductive costs for the aposematic moth *Arctia plantaginis*

**DOI:** 10.1093/beheco/araa033

**Published:** 2020-04-15

**Authors:** Carita Lindstedt, Kaisa Suisto, Emily Burdfield-Steel, Anne E Winters, Johanna Mappes

**Affiliations:** 1 Department of Biological and Environmental Sciences, University of Jyväskylä, Jyväskylä, Finland; 2 Institute for Biodiversity and Ecosystem Dynamics (IBED), University of Amsterdam, Amsterdam, The Netherlands

**Keywords:** chemical defense, color polymorphism, cost of defense, heritability

## Abstract

To understand how variation in warning displays evolves and is maintained, we need to understand not only how perceivers of these traits select color and toxicity but also the sources of the genetic and phenotypic variation exposed to selection by them. We studied these aspects in the wood tiger moth *Arctia plantaginis,* which has two locally co-occurring male color morphs in Europe: yellow and white. When threatened, both morphs produce defensive secretions from their abdomen and from thoracic glands. Abdominal fluid has shown to be more important against invertebrate predators than avian predators, and the defensive secretion of the yellow morph is more effective against ants. Here, we focused on the morph-linked reproductive costs of secretion of the abdominal fluid and quantified the proportion of phenotypic and genetic variation in it. We hypothesized that, if yellow males pay higher reproductive costs for their more effective aposematic display, the subsequent higher mating success of white males could offer one explanation for the maintenance of the polymorphism. We first found that the heritable variation in the quantity of abdominal secretion was very low (*h*^2^ = 0.006) and the quantity of defensive secretion was not dependent on the male morph. Second, deploying the abdominal defensive secretion decreased the reproductive output of both color morphs equally. This suggests that potential costs of pigment production and chemical defense against invertebrates are not linked in *A. plantaginis*. Furthermore, our results indicate that environmentally induced variation in chemical defense can alter an individual’s fitness significantly.

## Teaser

How much does it cost to defend against predators? A lot if you are a male wood tiger moth as deploying their defensive secretion decreases their reproductive success. When threatened, these aposematic moths produce defensive secretions from their abdomen, which are targeted specifically against invertebrate predators. However, this invertebrate-specific defense does not come without costs: deploying the defensive secretion only once decreases the reproductive success of males.

## INTRODUCTION

In aposematic prey species, individuals possess secondary defenses, such as toxins, coupled with warning signals. Warning signals are often in the form of conspicuous coloration, which advertises those defenses to potential predators (Ruxton et al. 2004). Conspicuous colors are expected to be favored over dull color patterns because predators associate a conspicuous signal with unpalatability more readily ( Gittleman and Harvey 1980; Gamberale-Stille and Tullberg 1999; Lindström et al. 1999). Increasing unprofitability of the prey also enhances avoidance learning in predators (Leimar et al. 1986; Ihalainen et al. 2007; Rowland et al. 2007). However, depending on the perceivers of these traits, their relative importance can vary, resulting in variation in aposematic coloration and secondary defenses (Bowers and Stamp 1997; Endler and Mappes 2004; Nokelainen et al. 2014; Briolat et al. 2018). For example, whereas avian predators are likely to be deterred by aposematic coloration, the strength of chemical or physical defenses can be more important against nonvisual predators, such as ants (Codella and Raffa 1995; Dyer 1995; Sugiura and Yamazaki 2014). The mechanisms behind unprofitability (e.g., distastefulness and toxicity) can also vary, resulting in different levels of protection within and among chemically defended species that share a similar appearance (Skelhorn and Rowe 2010; Holen 2013; Winters et al. 2018).

One potential outcome of selection by a variable predator community structure is strategies where animals have evolved multiple defensive responses that are targeted against different types of predators (Bowers and Stamp 1997; Rojas et al. 2017). However, producing these multiple chemical defense traits, as well as pigmentation for the warning signals, can be costly and they may compete for resources within an individual (Blount et al. 2009). For example, the production and maintenance of effective warning signals can decrease growth rate (Grill and Moore 1998), longevity (Ohsaki 2005), and immune defense (Friman et al. 2009). Additionally, certain pigment molecules used in warning colors can act as antioxidants (McGraw 2005; Dhinaut et al. 2017; Koch et al. 2018); thus, investing in brighter coloration may make animals more susceptible to oxidative stress caused by defensive chemicals (Blount et al. 2009). In this case, pigmentation and chemical defense can evolve in a correlative manner due to physiological constraints (Blount et al. 2009).

Similar to warning signal pigmentation, the production and maintenance of chemical defenses can be costly, and the magnitude of these costs can depend on host plant quality (Reudler et al. 2015; Lindstedt et al. 2018) and nutritional conditions (Grill and Moore 1998; Burdfield-Steel et al. 2018), which may also affect warning signal expression (Lindstedt et al. 2010; Blount et al. 2012). In the case of species that possess different forms of defense targeted toward different predator classes, resource allocation between signal and defense may be even more complex as specific signals and defenses may not be targeted toward the same predators (Rojas et al. 2017). In such cases, different predator types may create disruptive selection, with some forms of predation favoring investment in one aspect of defense while others favor different aspects. In this instance, understanding the costs associated with a particular aposematic trait becomes even more vital to accurately predict how different defensive traits are selected under different ecological conditions. This also requires the determination of the extent of heritable variation in a defensive trait and whether it is genetically correlated with other traits as it can affect the strength of evolutionary responses to directional selection by predators (Lindstedt et al. 2016).

**Figure 1:**
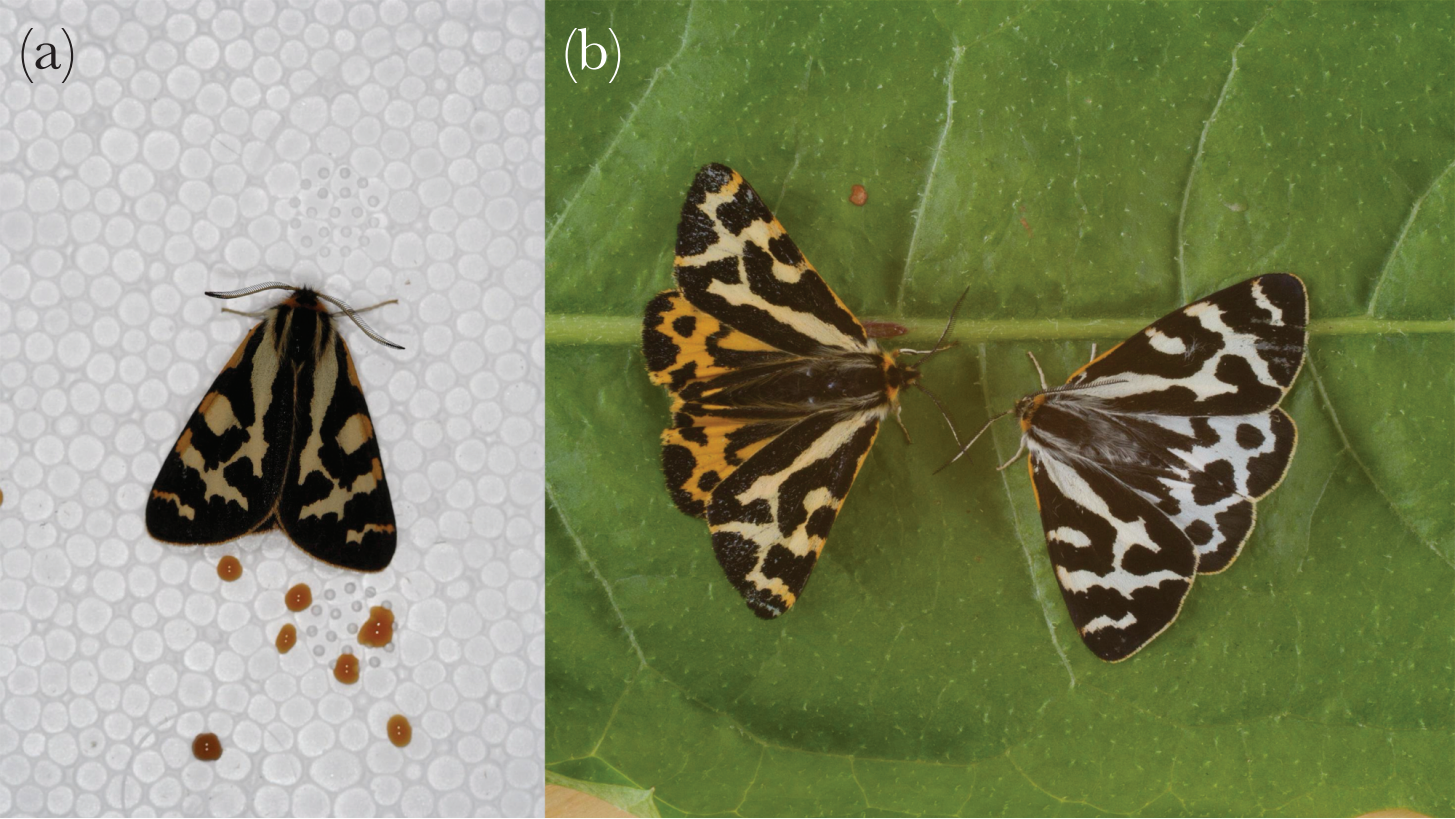
(a) *Arctia plantaginis* can produce defensive secretion from abdomen (Image: Janne Valkonen). (b) Yellow and white *A. plantaginis* male morphs (Image: Samuel Waldron).

The wood tiger moth, *Arctia plantaginis* (formerly *Parasemia plantaginis*), Arctiidae is an example of an aposematic species with a varied defensive repertoire against predators (Rojas et al. 2017): adult *A. plantaginis* moths can produce two types of secretions when disturbed by predators: one from the abdomen and one from the thoracic glands that differ in their chemical properties and deterrence against different types of predators (Rojas et al. 2017). The abdominal secretion (Figure 1a) is more effective against invertebrate predators, such as ants, and the secretion from the thoracic glands is targeted toward avian predators (Rojas et al. 2017).

In general, the defensive secretions are thought to function as short-term responses to immediate predatory threats (Wiklund and Järvi 1982; Skelhorn and Rowe 2006) and the animal most likely loses the excretion as a result of the defensive act, decreasing the individual’s defense capacity until the depleted resource has been replenished. In *A. plantaginis,* adults tend to release the abdominal defensive fluid under threat if they still have it but, because they do not feed it, it is likely that they can deploy each defense only a limited number of times (see Supplementary Information). We can expect that releasing a higher volume of deterrent fluid should increase the moth’s defense efficacy against invertebrate predators as it is likely to contain higher quantities of defensive compounds offering a stronger stimulus for predators (e.g., Lindstedt et al. 2017). In addition, releasing higher quantities of defensive secretion can be especially beneficial to defend against attacks by ants, which are more likely to prey upon the moths collectively (i.e., several individuals attacking at the same time).

However, releasing defensive secretions can be costly (Higginson et al. 2011; Lindstedt et al. 2018) and these costs can depend on the warning color pigmentation (Blount et al. 2009, 2012). In *A. plantaginis,* previous studies have shown that yellow males are more likely to survive predation attempts by avian predators (Nokelainen et al. 2012, 2014) even though efficacy of white and yellow warning coloration in males also varies depending on the predator community structure (Nokelainen et al. 2014). Furthermore, the abdominal defensive secretion of yellow males is more deterrent against ants than secretions from white individuals (Rojas et al. 2017). Tests of the defensive secretions from the neck glands of the yellow males, in the absence of color cues, suggest that they have a more repulsive odor against avian predators than those from whites. However, secretions from white males are more often taste rejected by birds, suggesting that the overall “strength” of these defenses may vary depending on the behavioral measure used. Therefore, on balance, we can expect that the selection from visual predators may vary (Holen 2013; Rojas et al. 2019) but, when combined with selection from invertebrate predators, we still expect males with yellow pigmentation to be favored overall by predation.

In this study, we had two aims: our first aim was to quantify the extent of individual variation susceptible to selection by invertebrate predators in the volumes of defensive secretions both in wild populations and under standardized laboratory conditions. We also determined how much of this variation is heritable in order to predict the evolutionary response of this chemical defense trait from one generation to the next under selection by predators. Higher heritability estimates indicate stronger potential responses to selection (Falconer and Mackay 1996).

Our second aim was to examine if deploying the defensive secretion is costly for an individual and whether these associated costs vary between white and yellow male morphs (Figure 1b). To measure the costs of responsive defense against invertebrate predators, we followed the reproductive success of males that were forced to deploy their abdominal secretion before mating and compared them to those of nonmanipulated individuals for both white and yellow male morphs. We assumed that, if investment in the more-effective predator defense in yellow males trades off with the reproduction, we should see differences in the fitness costs of chemical defense between white and yellow males (Nokelainen et al. 2012). Alternatively, it is possible that the production of a conspicuous warning color pattern and efficient chemical defense do not incur any costs (Ojala et al. 2007; Lindstedt et al. 2010, 2016). In this case, we should not detect any interactive effects of male color and fitness costs of defense (Lindstedt et al. 2010). To study if white males can compensate for the lower deterrence of their secretion by producing more (Rojas et al. 2017), we quantified the volume of abdominal secretions produced by white and yellow males to test if morphs differ in volumes produced under attack.

## MATERIAL AND METHODS

### Study species *A. plantaginis*

The larvae of the wood tiger moth, *A. plantaginis* (Arctiidae), are polyphagous and feed on numerous herbaceous and arborescent plant species (Ojala et al. 2005). Arctiid moths are capital breeders, that is, the adults do not feed. Individuals in this study originated from a laboratory stock reared under constant temperature (day: 22 °C; night: 18 °C) and density (20 larvae per family per container). This stock was founded using 50 wild pairs collected from central and southern Finland during summer 2010. The quantity of the abdominal fluid and the costs of producing the fluid were measured from individuals from the sixth generation. The laboratory population was complemented yearly with wild individuals collected from the same populations that founded the laboratory population. During the larval stage, food (dandelion, *Taraxacum sp*.) was offered ad libitum. The color of males (white or yellow) was determined by the eye (see Figure 1b for male morphs). However, classification of males to white and yellow morphs has been confirmed by spectrophotometer measurements, which showed clear differences based on reflectance (Nokelainen et al. 2012). Thus, both white and yellow male morphs are visually distinctive and easy to distinguish by the human eye and also in the eyes of likely bird predators (Nokelainen et al. 2012).

### Quantity of the chemical defense

To determine the phenotypic variation between white and yellow male moths in the volumes of abdominal defensive secretions produced and how it covaries with their performance, abdominal secretions were collected from 38 yellow and 45 white males from 30 different families (1–15 individuals per family). Before the experiment, adults were kept at +5 °C to keep them fresh from the day they hatched. We only used adults that had not released the secretion before. This was confirmed from the rearing containers (no signs of defense secretion spots). Released secretion is clearly visible even after drying (Figure 1a). The age of the individuals varied between 1 and 5 days. All the individuals were given the opportunity to drink water and warm up for 1 h before the measurements started. Two of the white males and five of the yellow males did not produce abdominal fluid. They were still included in the statistical analyses. Pupal mass was used as a measure of the moths’ size in statistical analyses.

Abdominal secretions were extracted from the individuals by lifting the moth by the wings with the soft flexible tweezers and gently squeezing the moth’s abdomen. The secretion produced was drawn into a capillary tube and the volume was measured. According to our supplementary data (Supplementary Figures S1 and S2), we can assume that the individuals deploy the majority of their abdominal secretion on the first attack (approximately 67% of the total quantity) and much lower quantities on the second or third attacks. Therefore, our sampling method, where the defensive secretion was measured only once per individual, captures most of the individual variation in the volumes susceptible to selection by predators. In addition, the method used to collect secretions only once was unlikely to physically harm individuals and potentially disturb their mating success.

We used a linear mixed model to test whether the amount of abdominal defense fluid differed between color morphs (white and yellow). Data for the quantity of defensive fluid were positively skewed and, thus, not normally distributed. We instead modeled them as gamma distributed with a log link. Because gamma distributions do not allow for 0 values, one decimal more than the precision of the data was added to the values referring to the quantity of defensive fluid (0.01). The volume of the secretion was set as the dependent variable and male color as the fixed factor. The size of the moth (pupal mass) and age were set as covariates in the final model and family as a random factor. All the analyses were conducted with R-studio (Version 1.1.419, 2009–2018 RStudio and packages “lmer” and “car”).

### Heritability estimates

To examine the heritability in the quantity of defensive secretion released, we measured the amounts of secretion moths produced using similar methods as described above. The pedigrees of *A. plantaginis* included two generations from the lab stock. In the first generation, we collected the secretions from 127 moths from 59 families and, in the second generation, from 83 individuals from 31 families. Heritability was estimated from pedigrees based on the individuals from generations 5 and 6. We only included individuals who produced fluid for the analyses because potentially nil individuals (intact individuals who potentially are not “willing” to produce fluid at all) were not separated from individuals that had already released the fluid before the measurements (and might not have fluid left to release). However, based on the life-history data, the proportion of nil individuals is likely to be very small (see above). During laboratory rearing, the effective population size was kept as large as possible to maintain genetic variation.

The genotypic and phenotypic variances of volume of defensive secretions were estimated by fitting a Bayesian model with the “brms” 2.9.0 R package (Bürkner 2018). Secretion volume was the response variable and pupal mass a fixed effect, and animal and dam effects were fitted as intercept random effects. The response variable was assumed to have a skewed normal distribution. Pedigree relatedness was used to model the covariance of the animal effects to allow estimating additive genetic variance. Priors for intercept and pupal mass effect were normally distributed with a mean of 0 and standard deviation (SD) of 10, priors for all SDs were half *t*-distributed with 3 degrees of freedom (df), location 0, and scale 20. Markov Chain Monte–Carlo estimation, using Hamiltonian Monte Carlo sampling, was run first for 5000 warm-up iterations and, then, 10 000 sampling iterations using four chains. We monitored chain convergence by trace plots (Supplementary Figure S3) and values, which were 1 for all values (i.e., model converged). We included pupa mass in the model as the size of the individual can affect the volume of defensive secretions (Lindstedt et al. 2018). Additive and maternal variance were obtained from posterior distributions of animal and dam effects, respectively, and narrow sense heritability for volume of defensive secretion was calculated as *h*^2^ = σ2A/(σ2A + σ2M + σ2E). We considered parameter values to be different if their 95% highest posterior density (HPD) intervals did not overlap.

### Wild population collection

To understand the extent of expressed phenotypic variation in abdominal defensive secretion in the wild and whether it reflects the variation observed under laboratory conditions, we collected samples from wild-caught white and yellow individuals and recorded if they had retained the fluid and how much they produced under simulated attack. Wild moths were collected from sites in Southern and Central Finland during summer 2014. Both males and females were caught with nets and males were collected using traps bated with lab-reared females. Once caught, moths were taken to Jyväskylä University and stored at +5 °C. One hour prior to sampling for fluids, they were moved to room temperature and provided with water. The secretion was collected as described above. The proportion of white and yellow males producing the abdominal fluid was compared with a chi-squared test.

### Reproductive costs of chemical defense

To measure the reproductive costs of chemical defense, we used the same laboratory-reared individuals from the abdominal fluid measurement (i.e., individuals that were forced to deploy defensive fluid) in addition to control individuals. On the same day after the fluid collection, we randomly chose 29 white males and 28 yellow males from 23 different families for the life-history measurements and mated them with randomly chosen, nonmanipulated, females. As a control, we mated 21 white and 18 yellow nonmanipulated males that had not been forced to produce the defense fluid and whose rearing containers showed no signs of previous defense. Reproductive assays for the nondepleted individuals were done at the same time as the depleted individuals. We recorded the number of eggs the females produced on the fourth day after egg-laying started. Larvae were counted on the seventh day after first hatching to make sure that all viable larvae had hatched. From these records, we calculated the hatching success (number of larvae/number of eggs).

We used linear mixed models to analyze whether defense fluid production influences reproductive success. We had number of eggs female produced and hatching success as dependent variables and male morph, treatment (forced defense), and their interaction as fixed factors and male’s family as a random factor. Female pupa mass, male pupa mass, and age were set as covariates. The interaction between male color and depletion treatment was not significant for either of the reproductive output traits (all *P* values >0.685) and were, therefore, omitted from the final models. The Satterthwaite approximation for degrees of freedom was applied when using the function “lmer.” Males’ likelihood to reproduce (female produced offspring or not) was treated as a dichotomous variable modeled as a binomial response variable with a logit link function.

## RESULTS

### Genetic and phenotypic variation in the quantity of abdominal secretion

**Figure 2:**
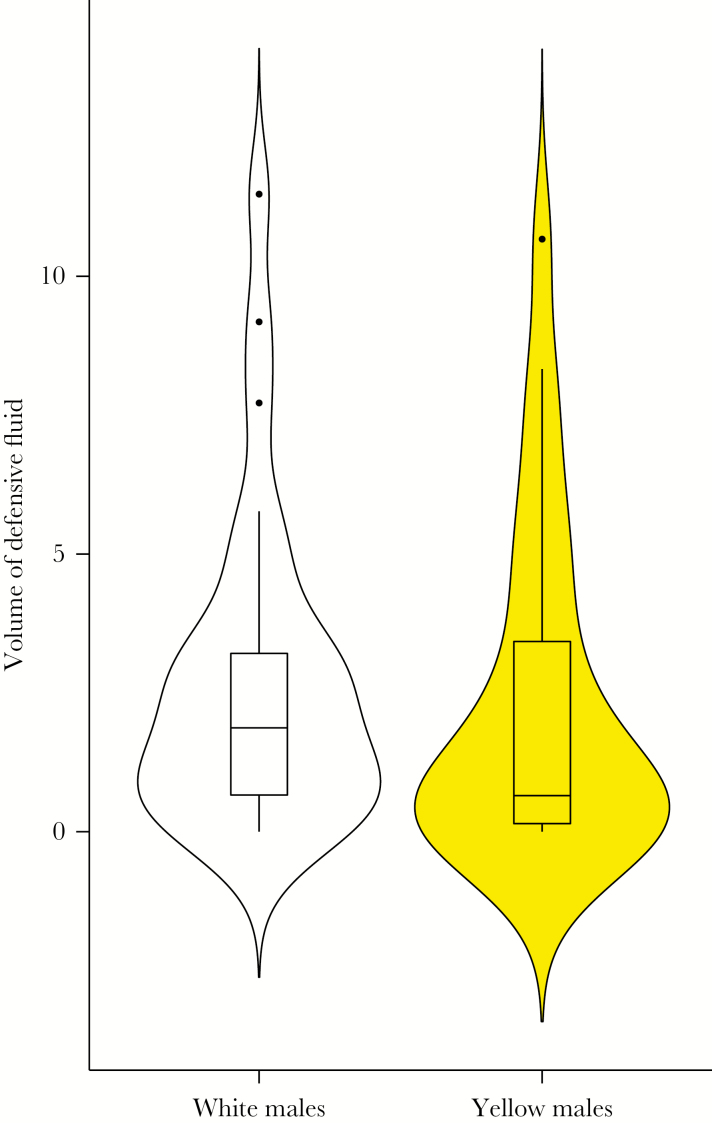
The volume (microliters) of abdominal defensive secretion of the two male color morphs (white and yellow) of wood tiger moths presented as violin plots overlaid with box plots. The violin plot outlines illustrate kernel probability density (i.e., the width of the area represents the proportion of the data located there).

The volume of abdominal secretion produced by laboratory-reared moths varied from 0 to 28.0 µL (mean: 6.14, standard error [SE]: ±6.6). The male morphs did not differ in the quantity they secreted (effect: −0.137, SE = 0.390, *t* = −0.352, *P* = 0.725; Figure 2). Age and pupal weight had no effect on the volume of secretion (male age: effect: −0.049, SE = 0.130, *t* = −0.377, *P* = 0.706; male pupa mass: effect: −0.519, SE = 0.786, *t* = −0.659, *P* = 0.510). We did not find significant heritable variation in the quantity of the defense fluid (*h*^2^ = 0.006 [0, 0.06], 95% HPD), additive variance (median 0.22 [0, 1.95], 95% HPD); maternal variance (median 0.23 [0, 2.01], 95% HPD), and environmental (residual) variance (median 34.51 [25.27, 45.39], 95% HPD).

A total of 64 wild male moths were collected and sampled from Southern and Central Finland, 16 yellow and 50 white. Approximately, half of them produced abdominal fluids (Table 1). There were no differences between white and yellow males in the probability to produce the abdominal secretion (*X*^2^ = 2.5, df = 3, *P* > 0.95). Wild yellow and white males did not differ significantly in the volumes they produced (*F*_1,61_ = 1.277, *P* = 0.263).

**Table 1:** Number and proportion producing abdominal fluids of wild moths samples in Central and Southern Finland in 2014

Population	Total number of moths sampled	Proportion producing abdominal fluid	Proportion of whites producing abdominal fluid	Mean ± SD volume produced by whites	Proportion of yellows producing abdominal fluids	Mean ± SD volume produced by yellows
Central Finland	16	0.56	0.50 (*N* = 9)	4.8 ± 4.1 µL	0.67 (*N* = 6)	1.5 ± 0.9 µL
Southern Finland	50	0.52	0.55 (*N* = 41)	2.2 ± 3.7 µL	0.38 (*N* = 9)	0.4 ± 0.2 µL
Combined	66	0.52	0.54 (*N* = 51)	2.8 ± 3.9 µL	0.50 (*N* = 15)	1.1 ± 0.9 µL

### Fitness costs of chemical defense

Males that had retained their defensive fluid were more likely to produce offspring than males that had deployed their defensive fluid (effect: −1.436, SE = 0.605, *z* = −2.375, *P* = 0.018). Males with higher pupa mass were more likely to produce offspring than males with lower pupa mass (effect: 2.119, SE = 0.921, *z* = 2.301, *P* = 0.021). Male color did not have a significant effect on reproductive success (effect: −0.855, SE = 0.533, *z* = −1.604, *P* = 0.109), neither did the male’s age (effect: −0.147, SE = 0.168, *z* = −0.875, *P* = 0.382).

**Figure 3:**
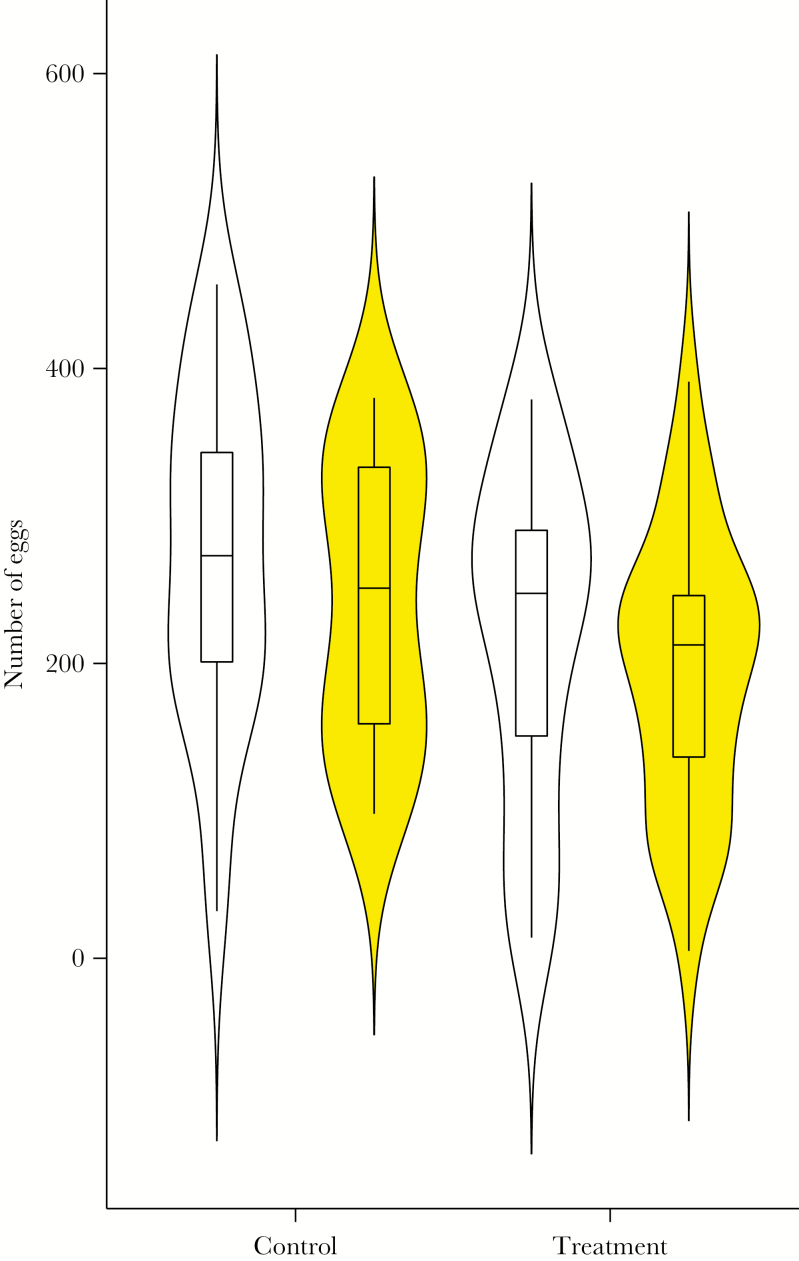
Egg number produced by females mated with either white or yellow male morphs presented as violin plots overlaid with box plots. Both morphs (yellow and white) were either forced to release abdominal defensive fluid (manipulation) or not manipulated (control).

Overall, females that mated with males that had been forced to release abdominal secretions produced fewer eggs than females that mated with the nonmanipulated control males (effect: −43.67, SE = 20.89; *F*_1,84_ = 4.368, *P* = 0.040; Figure 3). Female (effect: 136.17, SE = 31.31; *F*_1,84_ = 18.918, *P* < 0.001) and male pupa mass (effect: 67.74, SE = 33.86; *F*_1,84_ = 4.001, *P* = 0.049) both affected the total number of eggs produced. Individuals with higher pupa mass produced more eggs. The color of the male moth did not affect the egg number (effect = −32.81, SE = 20.89; *F*_1,83_ = 2.468, *P* = 0.120).

The depletion treatment (effect: −0.118, SE = 0.077; *F*_1,75_ = 2.270, *P* = 0.136) and male color (effect = −0.010, SE = 0.078; *F*_1,57_ = 0.015, *P* = 0.904) did not significantly affect hatching success. Nor was it significantly affected by female (effect: 0.197, SE = 0.115; *F*_1,85_ = 2.917, *P* = 0.091) or male pupa mass (effect: 0.110, SE = 0.122; *F*_1,73_ = 0.803, *P* = 0.373). Treatment (effect: −37.12, SE = 22.17; *F*_1,87_ = 2.761, *P* = 0.100) or male color (effect = −1.116, SE = 21.844; *F*_1,87_ = 0.003, *P* = 0.959) did not have a significant effect on the offspring number, but females with higher pupa mass produced a higher number of offspring (effect: 133.40, SE = 33.05; *F*_1,87_ = 16.077, *P* <0.001).

## DISCUSSION

To define how aposematic traits evolve, we need to understand the inheritance of these traits, the benefits of these traits against predators, and how costly they are to produce (Ojala et al. 2007; Speed and Ruxton 2007; Lindstedt et al. 2009, 2016). Here, we focused on two of these aspects: we, first, show that the volume of secreted defensive fluid targeted toward invertebrate predators varies extensively among individuals, but only a very low proportion of this variation is heritable suggesting weak responses to selection by predators. However, deploying this chemical defense against invertebrate predators incurs high life-history costs for *A. plantaginis* males, decreasing their reproductive output significantly. These costs were not dependent on the male color morph. Yellow males, who are better defended against multiple predators in terms of signal efficacy and the deterrence of their defensive fluid against invertebrate predators, did not pay higher life-history costs of chemical defense in comparison to white males. Therefore, color polymorphism in this species is not likely to be maintained via higher defensive costs in either of the male morphs.

Higher volumes of defensive fluid are often expected to ensure better defensive efficacy against predators (de Jong et al. 1991; Daly et al. 2012; Lindstedt et al. 2017; 2018) and, therefore, evolve under directional selection by predation decreasing variation in toxicity (Leimar et al. 1986; Ihalainen et al. 2007; Rowland et al. 2007). However, only a few studies have quantified the proportion of heritable variation in chemical defense traits that could respond to directional selection (Holloway et al. 1993; Speed et al. 2012). In *A. plantaginis*, the heritable variation in the volumes of fluids deployed was very low. Some of this might be explained by the moderate sample size for the heritability analyses. Nevertheless, based on these estimates, we cannot expect strong responses to selection toward the higher volumes of deployed fluid. Instead, continuous variation in the quantities of abdominal secretion can be a result of stochastic environmentally induced variation due to, for example, the deterred attack in the past or an individual’s condition (e.g., conditions experienced during the larval stage; Speed et al. 2012).

Our results from the phenotypic data collected from the wild-caught male moths are congruent with the results from the laboratory experiments. There were no differences between the white and yellow males in the frequency of depleted and nondepleted individuals. Nor did the volumes produced by the two morphs differ, supporting the findings of the laboratory experiments. Data collected from the wild populations also suggest that abdominal fluid is not something that individuals automatically lose during eclosion but rather that they are likely to retain it until needed for antipredator defense. Unfortunately, our field data does not tell us whether the wild individuals who did not produce the fluid had already mated or not. Still, if the loss of abdominal secretion has similarly enduring effects on mating success as we observed in the laboratory experiment, the ability to retain defensive fluid, and attack intensity by predators leading to the loss of the fluid before mating, could indirectly alter individuals’ reproductive success in the wild.

At present, we do not have any precise mechanism explaining the lower reproductive output of depleted individuals. The abdominal secretion is thought to be predominately comprised of waste product, but its water content can have a significant effect on the performance of adult individuals and may affect, for example, the amount of nutrients and water that females receive from the male via spermatophore. Even though a previous study did not reveal any differences in the fertility or spermatophore size between white and yellow males (Chargé et al. 2016), we do not know if the quality or size of the spermatophore changes due to depletion of the defensive fluids. In this case, a low amount of nutrients or water transferred with the spermatophore might lead to the production of fewer eggs by females but not necessarily decrease the fertility of the sperm. This explanation is supported by our results as we found decreased egg production in females but no differences in hatching success. On the other hand, a recent study shows that multiple mating, where females are likely to receive more nutrients and water during the mating, does not increase egg production or female longevity in this species (Santostefano et al. 2018). Additionally, the production of defensive secretions may be costly simply in terms of energy (e.g., the metabolic processes required to synthesize and expel the fluid), decreasing an individual’s performance and reproductive output (Higginson et al. 2011; Lindstedt et al. 2018). Therefore, allocating resources to defense could trade off with other traits that increase lifetime fitness, such as survival and quality as a mate.

Experiments in more natural conditions where females have had a choice between several males suggest that variation in the reproductive success of white and yellow males is likely to be more complex and vary depending on the frequency of the male morphs (Gordon et al. 2015). Gordon et al. showed that the more frequent morph (white or yellow) had an advantage in mating success, but this advantage disappeared when the frequency of white and yellow males was more balanced (Gordon et al. 2015). In future research, it would be important to test the effect of retaining the abdominal fluid until the mating is in a more natural setup where female choice and possible male–male competition between white and yellow males is not excluded. For example, in a previous experiment by (Nokelainen et al. 2012), white and yellow males differed in their probability to mate, which we did not measure here. We could expect to find lower mating success for depleted individuals but potentially also differences between yellow and white males that are not necessarily visible under laboratory conditions in no-choice mating experiments (Nokelainen et al. 2012; Gordon et al. 2015; Dougherty 2020).

Another interesting avenue for future research will be to study more precisely the phenotypic and genetic variation among individuals who produce or do not produce abdominal fluid at all (see also, e.g., Lindstedt et al. 2018). Even though the proportion of those individuals was relatively small in our data sets (see also Supplementary Material), it would be interesting to disentangle if those nil individuals prioritize their reproduction over invertebrate defense or if they are simply unable to deploy the defense (do not have fluid or have already lost it). It is also important to note that our measurements here provide information about the quantities that individuals are “willing” to release to defend themselves when threatened by a predator. This most likely represents the variation predators experience when attacking the moth. However, to reliably measure the absolute quantity of defensive secretion reserves, we must first locate the defensive glands or stores and dissect them (e.g, Lindstedt et al. 2018). After a dissection, it is no longer possible to measure the subsequent life history and adult reproductive traits that were of interest in this study.

In conclusion, there is accumulating evidence that deploying defensive secretions incurs life-history costs for a prey individual decreasing growth, lifespan, and reproductive output. In many of these studies, these effects are based on the repetitive production of defensive response (Higginson et al. 2011; Lindstedt et al. 2018). Our study here shows that deploying this kind of responsive defense only once can still be highly costly for an individual in terms of reproductive success. Our results also suggest that when chemical defense is predominantly environmentally determined, explaining the maintenance of its diversity becomes, perhaps, a little less paradoxical (Speed et al. 2012).

## FUNDING

This study was funded by the Academy of Finland via Centre of Excellence in Biological Interactions grant (no 252411) and individual grants (no 136387, 257581) for C.L.

We are grateful to Ilkka Kronholm for valuable help with the heritability analyses, all the research assistants helping to maintain insect cultures, and the *Ecology and Evolution Journal* club for commenting on earlier versions of the manuscript.

Data accessibility: Analyses reported in this article can be reproduced using the data sets provided by Lindstedt et al. 2020.

## Supplementary Material

araa033_suppl_Suppelementary_Material
