# Supplementary material for: Defense against predators incurs high reproductive costs for the aposematic moth Arctia plantaginis
Source: Behav Ecol. 2020 Apr 15;31(3):844–50. doi: 10.1093/beheco/araa033 (PMC7303824; doi:10.1093/beheco/araa033)
Supplement: araa033_suppl_Suppelementary_Material [file araa033_suppl_suppelementary_material.docx]

Supplementary information

**Defence against predators incurs high reproductive costs for the aposematic moth *Arctia plantaginis***

*Carita Lindstedt^1^, *Kaisa Suisto^1^, Emily Burdfield-Steel^1,2^, Anne Winters^1^, Johanna Mappes^1^

*shared first authorship

1) Department of Biological and Environmental Sciences, University of Jyväskylä, Finland

2) Institute for Biodiversity and Ecosystem Dynamics, University of Amsterdam, The Netherlands

**Corresponding author:**

Carita Lindstedt

Department of Biological and Environmental Sciences,

University of Jyväskylä, P.O. Box 35,

FI-40014 University of Jyväskylä, Finland

carita.a.lindstedt@jyu.fi

**Defensive behaviour of *Arctia plantaginis* male moths**

To study the phenotypic and genetic variation in the volumes of abdominal defensive secretion released by *Arctia plantaginis* male moths, we measured the quantity of defensive secretion only once per individual. Therefore, to further confirm if this method reliably captures the variation in the quantity of fluid released by males, we collected an additional data, where we depleted laboratory reared males repeatedly (once per day over three consecutive days) and measured 1) how many individuals deployed the fluid for the first time for each attack (moth was lifted from the wings and gently squeezed with soft forceps from the abdomen), 2) the volume of fluid they released on the first, second and third attack (no fluid released = 0) and 3) how much fluid they released on the first, second, and third deployment (males who did not produce fluid were excluded). We used the second generation of laboratory reared males from 2019 who originated from the first generation of wild-caught females collected from the Central Finland. Individuals were reared in a standardized conditions similar to described in the methods section. Altogether we collected 27 yellow males and 50 white males.

We used linear mixed models to analyse whether the volume of fluid released depends on 1) how many times the moth has been attacked or 2) how many times it has deployed the fluid. We also included colour morph and pupal weight as dependent variables together with individual ID nested within family as a random factor. We used a Tukey-Kramer HSD *post hoc* test to interpret significant differences within the three levels of attack or deployment. All analyses were conducted with R version 3.6.1.

We found that 78% of individuals deployed the fluid already on the first attack (i.e. first day), 8% deployed the fluid for the first time on the second attack (i.e. second day), none deployed the fluid for the first time on the third attack, and 14% never deployed the fluid. There was no effect of colour morph or pupal weight on the volume of defence fluid released over consecutive attacks (colour morph: χ^2^ = 0.0034, d.f. = 1, *p* = 0.9532; pupal weight: χ^2^ = 0.8871, d.f. =1, *p* = 0.3463) or over consecutive deployment (colour morph: χ^2^ = 0.1077, d.f. = 1, *p* = 0.7428; pupal weight: χ^2^ = 0.0164, d.f. = 1, *p* = 0.8982). There were differences in the volume released between the first, second, and third attack (χ^2^ = 36.48, d.f. = 2, *p* < 0.0001; Fig S1), with more fluid released (67% of the total fluid) on the first attack compared to the second (Tukey *post hoc* test, *p* < 0.0001, Fig S1) or third (Tukey *post hoc* test *p* < 0.0001, Fig S1). We also found differences in volume released between the first, second, and third deployment (χ^2^ = 20.28, d.f. = 2, *p* < 0.0001; Fig S2), with more fluid released (70% of the total fluid) on the first deployment compared to the second (Tukey *post hoc* test *p* = 0.0016; Fig S2) or third (Tukey *post hoc* test *p* = 0.0013; Fig S2).


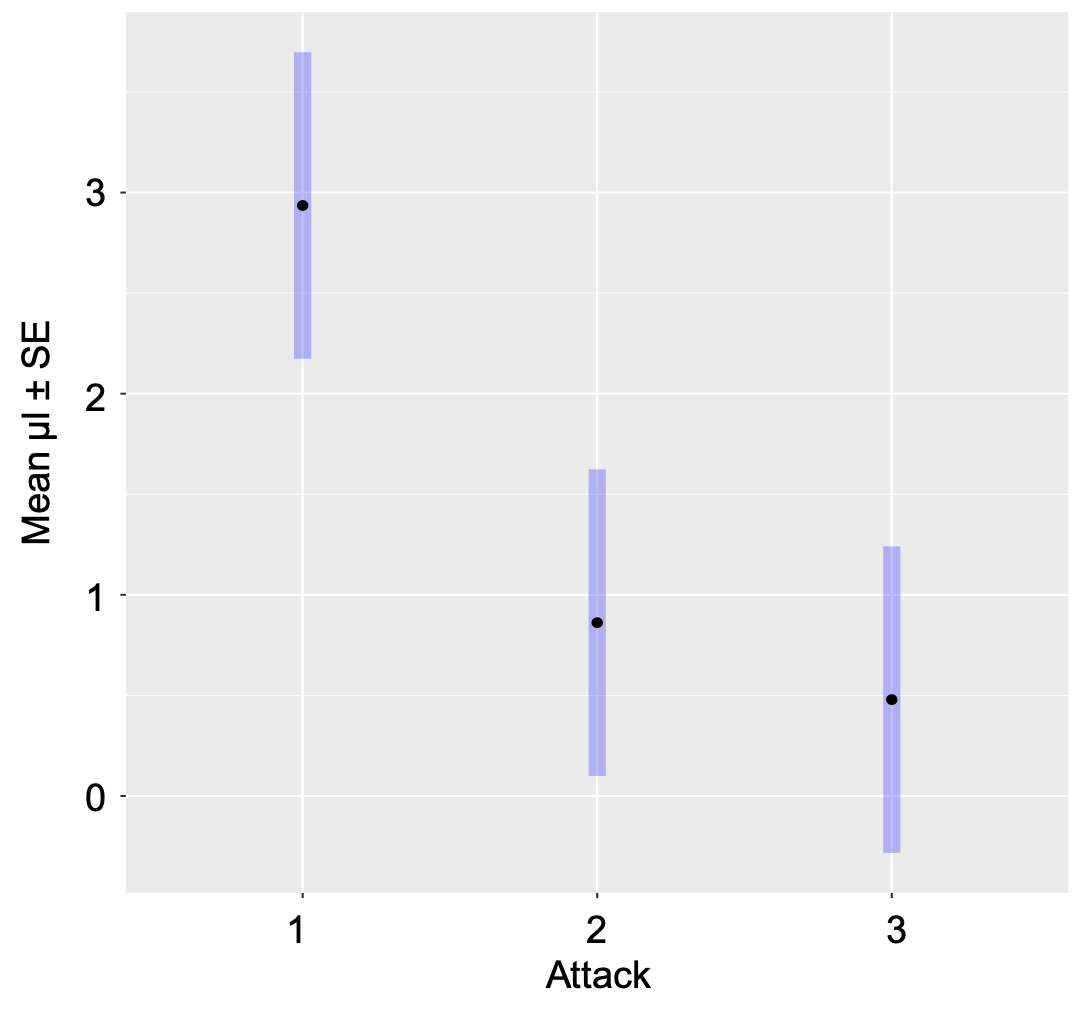


Fig S1. Mean μl of fluid released per moth for each of the three attacks. Bars indicate the standard error of the mean.


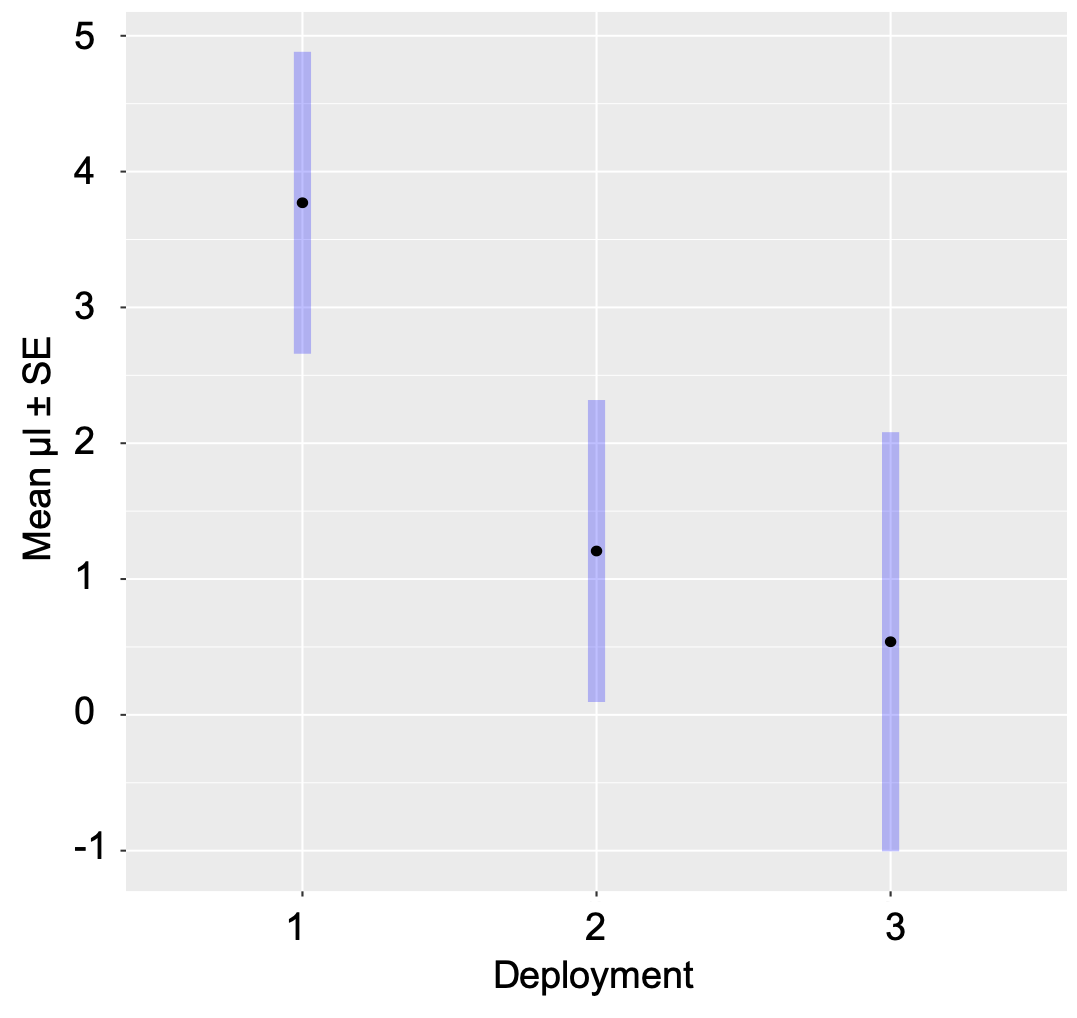


Fig S2. Mean μl of fluid released per moth each time fluid was deployed (males that produce no fluid were excluded). Bars indicate the standard error of the mean.

Figure S3. Trace plots to assess chain convergence in MCMC.
